# COVID-19 in pregnancy: A cross-sectional study on clinical features, disease severity, and health outcome

**DOI:** 10.17305/bb.2023.9748

**Published:** 2024-06-01

**Authors:** Rozhin Amin, Mohammad-Reza Sohrabi, Ali-Reza Zali, Khatereh Hannani

**Affiliations:** 1 Community Medicine Department, School of Medicine, Shahid Beheshti University of Medical Sciences, Tehran, Iran; 2 Social Determinants of Health Research Centre, Shahid Beheshti University of Medical Sciences, Tehran, Iran; 3 Functional Neurosurgery Research Centre, Shahid Beheshti University of Medical Sciences, Tehran, Iran; 4 Statistics and Information Technology Management, Shahid Beheshti University of Medical Sciences, Tehran, Iran

**Keywords:** Coronavirus disease 2019 (COVID-19), disease severity, Iran, pandemics, pregnant women, severe acute respiratory syndrome coronavirus 2 (SARS-CoV-2)

## Abstract

Assessing the impact of coronavirus disease 2019 (COVID-19) reveals unique challenges for pregnant women, who experience distinct clinical manifestations and health outcomes compared to their non-pregnant counterparts. We aimed to evaluate the clinical features, disease severity, and health outcomes of COVID-19 in pregnant women and compare them to those of non-pregnant women. In this population-based study, we included all women diagnosed with COVID-19 across the province of Tehran during the first two years of the epidemic. Descriptive statistics, the chi-squared test, and the logistic regression model were applied. Overall, 79,338 non-pregnant women and 3249 pregnant women diagnosed with COVID-19 were included. Pregnant women were most commonly in the age group of 25–34 years (54%, *n* ═ 1758), while the age group of 34–44 had the highest representation among non-pregnant women (56%, *n* ═ 44,492). After accounting for age and comorbidities, pregnancy was associated with an increased risk of requiring intensive care (odds ratio [OR] 1.38, confidence interval [CI] 1.223–1.564). However, the probability of dying due to severe acute respiratory syndrome coronavirus 2 (SARS-CoV-2) infection was lower in pregnant women compared to non-pregnant women (OR 0.55, CI 0.394–0.793). Cough (41%) and fever (30%) were the most frequent clinical presentations in pregnant women, whereas cough (57%) and muscle ache (38%) were the most common symptoms in non-pregnant women. Furthermore, diarrhea (*P <* 0.001) and skin lesions (*P <* 0.001) were reported more frequently by pregnant patients than non-pregnant patients. A significant prevalence of diabetes (*P <* 0.001), hypertension (*P <* 0.001), cancers (*P <* 0.001), and chronic hematological diseases (*P <* 0.001) was observed in pregnant patients. In conclusion, COVID-19-infected pregnant women exhibit different clinical manifestations and a more severe clinical course but have better health outcomes compared to their non-pregnant counterparts.

## Introduction

The new coronavirus, severe acute respiratory syndrome coronavirus 2 (SARS-CoV-2), was declared a global pandemic by the World Health Organization (WHO) in March 2020 [1, 2]. Since its declaration, the disease has significantly impacted lives worldwide, with over 750 million confirmed cases and approximately seven million deaths to date [3]. The first report of an outbreak in the Middle East and North Africa (MENA) region emanated from Iran, which confirmed two coronavirus disease 2019 (COVID-19)-related deaths in late February. Subsequently, Iran has experienced seven consecutive waves of the disease, with over seven million confirmed cases and about 145,000 deaths [4, 5].

As the pandemic continues, cases and casualties have been exponentially increasing, reflecting the nature of viral spread. While the virus affects all demographics, certain subpopulations are more susceptible to severe manifestations, leading to complications and death due to COVID-19 [6]. Pregnant women are among these high-risk groups, evidenced by their more adverse prognosis compared to non-pregnant individuals during previous viral epidemics such as severe acute respiratory syndrome coronavirus 1 (SARS-CoV-1), first reported in China in 2003, and Middle East respiratory syndrome coronavirus (MERS-CoV), initially identified in Saudi Arabia in 2012 [7]. Given the heightened vulnerability of pregnant women to similar viral infections, concerns about the effects of SARS-CoV-2 on this population have arisen, spurring numerous studies examining the health outcomes of pregnant patients diagnosed with COVID-19. Existing evidence indicates that pregnant women are more susceptible to severe illness following SARS-CoV-2 infection. Physiological changes during pregnancy render these patients more prone to intensive care unit (ICU) admissions when diagnosed with COVID-19 [8–10]. However, current literature lacks comprehensive clarity on the characteristics and outcomes for these patients. Moreover, most available data stem from case series or single-center studies with limited sample sizes [11]. As the pandemic progresses and more pregnant individuals contract COVID-19, there is a pressing need for population-based studies with reduced bias. These studies are pivotal for guiding policymakers and healthcare providers. Thus, this study aims to evaluate the clinical features, disease severity, and health outcomes of pregnant women with COVID-19 in comparison to non-pregnant infected women. For this purpose, we utilized data from a vast population, encompassing over 80,000 women of reproductive age diagnosed with SARS-CoV-2 during the first 20 months of the epidemic in Iran.

## Materials and methods

For this observational study, we extracted data on all women diagnosed with COVID-19 across the province of Tehran from March 2020 to October 2021 from the COVID-19 registry database. Tehran is the most populous province in Iran, with an estimated 14 million residents. This province has been a significant epicenter of the COVID-19 outbreak in Iran since the pandemic’s onset [12]. The COVID-19 registry was established in March 2020 documenting data on all patients diagnosed with COVID-19 across the province following the WHO case definition guidance [13]. In this descriptive multicenter study, we analyzed data from 82,587 women of reproductive age (15–44 years) diagnosed with SARS-CoV-2 infection. Out of these, 3249 (4%) were pregnant during their time of admission.

### Variables

Data related to age, smoking history, opioid use, underlying health conditions, clinical and paraclinical findings upon admission, ICU admission, and patients’ disease outcomes were obtained from the registry database. For clearer interpretation, variables were categorized as follows: (1) Age was divided into three categories: 15–24, 25–34, and 35–44 years. (2) History of smoking, opioid use, underlying health conditions, such as diabetes, hypertension, cardiovascular diseases (CVD), cancer, asthma, chronic liver diseases, chronic kidney diseases, chronic neurological conditions, chronic hematological diseases, and chronic immune deficiency disorders, were classified as either “yes” or “no.” (3) Diagnostic tests: Polymerase chain reaction (PCR) test results: Categorized into “positive” or “negative/inconclusive.” (4) Chest computed tomography (CT) reports: Classified based on findings related to COVID-19 (like patchy ground-glass opacities, crazy paving appearance, patchy consolidations with surrounding ground-glass halo, and patchy consolidations with or without an air-bronchogram) and reports without COVID-19-related findings. (5) Blood oxygen saturation level (PaO2): Dichotomized based on the National Coronavirus Control Protocol, using a cut-off value of 93% [14]. (6) ICU admission: Used as an indicator for disease severity, this variable was recorded as “yes” or “no.” (7) Disease outcome: Defined as “survived” or “deceased.”

### Ethical statement

Shahid Beheshti University of Medical Sciences Ethics Committee approved the study with a waiver of informed consent before the study began (reference number: IR.SBMU.RETECH.REC.1400.317). All data were de-identified prior to analysis.

### Statistical analysis

To capture the characteristics of the study population, descriptive statistics were employed. The chi-squared test was utilized to identify significant differences in the characteristics of pregnant and non-pregnant patients. To account for the potential confounding effects of age and existing comorbidities on disease severity and patient health outcomes, a logistic regression model was employed. Analyses were conducted using IBM SPSS Statistics, version 27 (IBM Corp., Armonk, NY, USA). A *P* value of 0.05 was designated as the level of significance. Throughout the province, data collection was streamlined using a standardized online form and carried out by trained healthcare personnel. Hence, the rate of missing information was low and its impact on statistical inferences could be considered insignificant.

## Results

In total, 79,338 non-pregnant women and 3249 (4%) pregnant women diagnosed with COVID-19 were included in this study. Pregnant women predominantly belonged to the age group of 25–34 years, while the 34–44 age group was the largest for non-pregnant women. There was no difference in smoking prevalence between the two groups. However, pregnant patients had higher instances of a positive history of opioid use. A greater proportion of pregnant patients reported diabetes, hypertension, cancers, and chronic hematological diseases. Nonetheless, conditions like CVD and chronic kidney diseases were more common in non-pregnant women. Cough and fever were the primary clinical symptoms in pregnant women, in contrast to cough and muscle aches in non-pregnant women. Generally, symptoms like fever, cough, muscle ache, breathing difficulties, chest pain, loss of taste, loss of smell, appetite loss, and vertigo were less common in pregnant women. On the other hand, diarrhea and skin lesions were more frequent in pregnant women. There was no significant difference in the prevalence of nausea, headaches, and seizures between the groups (Table 1).

**Table 1:** Characteristics of pregnant and non-pregnant women diagnosed with COVID-19, Tehran, 2020–2021

**Characteristics**		**Pregnant women**		**Non-pregnant women**		***P* value**
		***n***	**%**	***n***	**%**	
Age (years)	15–24	543	16.7	7965	10.0	<0.001
	25–34	1758	54.1	26,881	33.9	
	35–44	948	29.2	44,492	56.1	
Positive history of smoking		15	0.5	365	0.5	0.98
Positive history of opioids		17	0.5	147	0.2	<0.001
Underlying diseases	Diabetes	185	5.7	1790	2.3	<0.001
	Hypertension	88	2.7	1454	1.8	<0.001
	CVD	12	0.4	1280	1.6	<0.001
	Cancer	3	0.1	698	0.9	<0.001
	Asthma	26	1.0	629	1.0	0.79
	Chronic liver diseases	6	0.2	200	0.3	0.45
	Chronic kidney diseases	10	0.3	525	0.7	0.01
	Chronic neurological diseases	20	0.6	390	0.5	0.32
	Chronic immune deficiency diseases	8	0.2	287	0.4	0.27
	Chronic haematological diseases	35	1.1	341	0.4	<0.001
Clinical presentations	Fever	977	30.1	27,623	34.8	<0.001
	Cough	1357	41.8	45,691	57.6	<0.001
	Muscle ache	807	24.8	30,704	38.7	<0.001
	Difficulty breathing	808	24.9	27,893	35.2	<0.001
	Chest pain	62	2.0	2054	2.8	0.007
	Loss of smell	64	2.0	2689	3.4	<0.001
	Loss of taste	42	1.3	1674	2.1	0.001
	Loss of appetite	146	4.6	5718	7.6	<0.001
	Nausea	239	7.6	5686	7.6	0.97
	Diarrhea	156	4.9	2562	3.4	<0.001
	Headache	330	10.5	8560	11.6	0.07
	Vertigo	69	2.2	2395	3.2	0.001
	Seizure	8	0.2	147	0.2	0.43
	Paraplegia	0	0.0	50	0.1	0.14
	Skin lesions	10	0.3	82	0.1	<0.001

CVD: Cardiovascular diseases; COVID-19: Coronavirus disease 2019.

A higher number of pregnant women showed low blood oxygen saturation at admission compared to non-pregnant women. About 40% of patients in both groups were diagnosed based on positive PCR test results for SARS-CoV-2. For the rest, the diagnosis was determined by chest CT findings and clinical-epidemiological criteria. These criteria for COVID-19 diagnosis consider both clinical symptoms (like fever and cough) and epidemiological history (e.g., recent exposure to confirmed cases or travel to affected regions). Such criteria are vital for case identification and management, especially during outbreaks or in areas with limited testing resources [14]. Positive COVID-19 findings in chest CT reports were rarer in pregnant women. While a higher percentage of pregnant women needed intensive care, their mortality rate was lower compared to non-pregnant women (Table 2).

**Table 2:** Paraclinical findings and health outcome of pregnant and non-pregnant women diagnosed with COVID-19, Tehran, 2020–2021

**Characteristics**	**Pregnant women**		**Non-pregnant women**		***P* value**
	***n***	**%**	***n***	**%**	
PaO2 < 93%	2671	82.2	48,863	61.6	<0.001
Positive PCR test	1362	41.9	31,526	39.7	0.01
Chest CT with positive findings	818	25.2	48,783	61.5	<0.001
ICU admitted	308	9.5	5565	7.0	<0.001
Deceased	33	1.0	1591	2.0	<0.001

PCR: Polymerase-chain reaction; CT: Computed tomography; ICU: Intensive care unit; COVID-19: Coronavirus disease 2019.

Table 3 presents the results from the logistic regression analysis on independent variables related to COVID-19 severity. After adjusting for age and underlying conditions, being pregnant was linked to a higher risk of needing intensive care. When sorted by age, ICU admissions were least common in the 25–34 years age group, irrespective of pregnancy.

**Table 3:** Logistic regression analysis of independent variables associated with COVID-19 severity, Tehran, 2020–2021

**Variable**	**AOR**	**95% CI**		***P* value**
		**Lower**	**Upper**	
*Age group (years)*				
15–24	1			
25–34	0.89	0.814	0.981	0.01
35–44	0.93	0.854	1.020	0.12
*Pregnancy status*				
Non-pregnant women	1			
Pregnant women	1.38	1.223	1.564	<0.001
Positive history of smoking	1.50	1.092	2.065	0.01
Positive history of opioids	3.85	2.642	5.624	<0.001
Diabetes	2.31	2.043	2.630	<0.001
Hypertension	1.58	1.356	1.840	<0.001
CVD	2.93	2.546	3.385	<0.001
Cancer	3.53	2.933	4.252	<0.001
Asthma	1.48	1.158	1.904	0.002
Chronic liver diseases	2.70	1.920	3.799	<0.001
Chronic kidney diseases	2.50	2.003	3.123	<0.001
Chronic neurological diseases	2.83	2.193	3.652	<0.001
Chronic immune deficiency diseases	1.75	1.261	2.443	<0.001
Chronic haematological diseases	1.82	1.366	2.432	<0.001
Constant	0.07			

AOR: Adjusted odds ratio; CI: Confidence interval; CVD: Cardiovascular diseases; COVID-19: Coronavirus disease 2019.

After accounting for potential confounders such as age and other comorbidities in the logistic regression model, the risk of death due to SARS-CoV-2 infection was found to be lower in pregnant women compared to their non-pregnant counterparts (Table 4).

**Table 4:** Logistic regression analysis of independent variables associated with COVID-19 related death, Tehran, 2020–2021

**Variable**	**AOR**	**95% CI**		***P* value**
		**Lower**	**Upper**	
*Age group (years)*				
15–24	1			
25–34	0.89	0.739	1.089	0.27
35–44	1.36	1.142	1.633	<0.001
*Pregnancy status*				
Non-pregnant women	1			
Pregnant women	0.55	0.394	0.793	<0.001
Positive history of smoking	0.39	0.157	0.979	0.04
Positive history of opioids	4.27	2.245	8.140	<0.001
Diabetes	2.14	1.726	2.647	<0.001
Hypertension	1.65	1.287	2.132	<0.001
CVD	1.69	1.285	2.239	<0.001
Cancer	7.08	5.632	8.918	<0.001
Asthma	1.67	1.098	2.541	<0.001
Chronic liver diseases	3.47	2.154	5.618	<0.001
Chronic kidney diseases	4.81	3.626	6.400	<0.001
Chronic neurological diseases	2.80	1.826	4.308	<0.001
Chronic immune deficiency diseases	2.09	1.281	3.419	0.003
Chronic haematological diseases	2.90	1.948	4.325	<0.001
Constant	0.15			

AOR: Adjusted odds ratio; CI: Confidence interval; CVD: Cardiovascular diseases; COVID-19: Coronavirus disease 2019.

## Discussion

This population-based study provides a descriptive analysis of clinical features and health outcomes for 3249 pregnant and 79,338 non-pregnant women diagnosed with COVID-19 in Iran during the first 20 months of the pandemic. Our findings suggest that pregnant patients had a higher likelihood of ICU admission than non-pregnant patients. However, the probability of mortality due to COVID-19 was lower among pregnant women. In terms of symptoms, fever, muscle ache, respiratory, and neurological presentations were less prevalent in pregnant women. Conversely, diarrhea and skin lesions were reported more frequently in this group compared to their non-pregnant counterparts. Pregnant women infected with COVID-19 appeared to experience a more severe clinical course than their age-matched counterparts. After adjusting for age and underlying conditions, our results revealed that pregnant women were 1.3 times more likely to require ICU admission, consistent with studies conducted in Iran and other locations [15–17]. Several factors might explain these findings, including the immunological changes during pregnancy. An elevated humoral immune response and suppressed cell-mediated immunity, which naturally occur in pregnant patients to prevent the rejection of fetal tissue, might alter the maternal immune response to viral infections, including COVID-19. Hence, pregnancy-induced immunological changes could predispose pregnant women who are infected with SARS-CoV-2 to manifest more severe forms of the disease [18]. In addition, recent studies have pointed out the important role of the renin-angiotensin system (RAS). During pregnancy, there is a natural shift in RAS from the angiotensin-converting enzyme (ACE)-angiotensin II (Ang II)-angiotensin II type 1 (AT1)-AngII-AT1 axis toward the ACE2-Ang-(1–7) axis which leads to increased vasodilation, reduced inflammation, and decreased thrombosis in pregnant women. The SARS-CoV-2 virus uses ACE2 receptors for entry into human cells. However, reduced ACE2 receptor expression due to viral adhesion can disrupt the balance between the ACE-AngII-AT1 axis and ACE2-Ang-(1–7) axis, leading to increased vasoconstriction, inflammation, thrombosis, and pulmonary damage [19]. The higher ICU admission rates for pregnant women might also reflect the lower threshold for admitting them to ICUs in many Iranian hospitals and in other countries.

The death rate of pregnant women documented in this study (1.0%) aligns closely with findings from India (1.01%) and the overall maternal mortality rate reported in a review examining data from 15 countries worldwide (1.13%) [15, 20]. While some suggest that pregnancy adversely affects the clinical progression of patients with COVID-19, outcomes seem to be more favorable in pregnant patients compared to their non-pregnant counterparts. When accounting for age and underlying health conditions, our results revealed that pregnant women were less likely to die as a result of SARS-CoV-2 infection. This finding was consistent with previous results in the literature and might partly be due to the clinical improvement observed in pregnant women with severe COVID-19 post-delivery [21–23]. Another factor might be the specialized care pregnant women receive. Typically, they are considered high-risk and are often subjected to multidisciplinary care with prompt referrals to senior clinicians when complications arise, leading to differential management compared to non-pregnant individuals. In terms of clinical symptoms, cough and fever were the most commonly reported in pregnant women, consistent with prior studies [24, 25]. Compared to non-pregnant women, the former reported fever, cough, muscle ache, difficulty breathing, chest pain, and loss of taste, smell, appetite, and sensations of vertigo less frequently. However, instances of diarrhea and skin lesions were higher. Our data suggests that COVID-19 symptoms can vary between pregnant and non-pregnant individuals. Still, research directly comparing these two groups remains limited, emphasizing a need for further investigation.

Regarding comorbidities, pregnant patients more frequently presented with diabetes, hypertension, cancer, and chronic hematological diseases. While the literature has established a link between these conditions and worse COVID-19 outcomes in pregnant patients [26], it remains uncertain if these conditions increase the risk of SARS-CoV-2 infection in this group. Notably, our study identified a significant difference in PCR positivity rates between pregnant and non-pregnant groups—an unexpected finding warranting further research. Future studies might also benefit from exploring the relationship between preeclampsia and COVID-19 outcomes in pregnancy, a topic beyond the scope of our current research.

Limitations of our study include the absence of data on pregnancy outcomes, such as preterm labor, delivery type, and birth weight, preventing us from assessing COVID-19’s impact on pregnancy and neonatal health. Due to testing policies, women with mild or no symptoms were typically not tested. Nonetheless, since this approach was consistent across both groups, its impact on our findings is likely minimal. A significant strength of our study lies in its foundation on real-time, consistent data from a large multicenter cohort of COVID-19 patients, making its insights valuable for policymakers and clinicians responsible for pregnant patient care.

## Conclusion

This study reveals that pregnant patients diagnosed with COVID-19 have a heightened risk of ICU admission compared to their non-pregnant counterparts. Intriguingly, the likelihood of succumbing to SARS-CoV-2 infection is lower for pregnant individuals. Additionally, our data suggests variations in the clinical manifestations of COVID-19 between pregnant and non-pregnant women. These insights bear significant implications for clinicians managing pregnant women with COVID-19 and policymakers. It is imperative to heighten awareness among pregnant women about their increased vulnerability to severe COVID-19 manifestations. Consequently, emphasis on preventive measures against SARS-CoV-2 infection for this demographic is of paramount importance.

## Data Availability

The data supporting the findings of this study are available from the corresponding author, upon request and with permission of the Coronavirus Control Operations Headquarter in Tehran.
